# Supplementary material for: Cyclophilin J limits inflammation through the blockage of ubiquitin chain sensing
Source: Nat Commun. 2018 Oct 22;9:4381. doi: 10.1038/s41467-018-06756-3 (PMC6197184; doi:10.1038/s41467-018-06756-3)
Supplement: Supplementary file 1 — Supplementary Information [file 41467_2018_6756_MOESM1_ESM.pdf]

Supplementary Information

Cyclophilin J limits inflammation through the blockage of ubiquitin chain sensing

Sheng et al.

Supplementary Figures

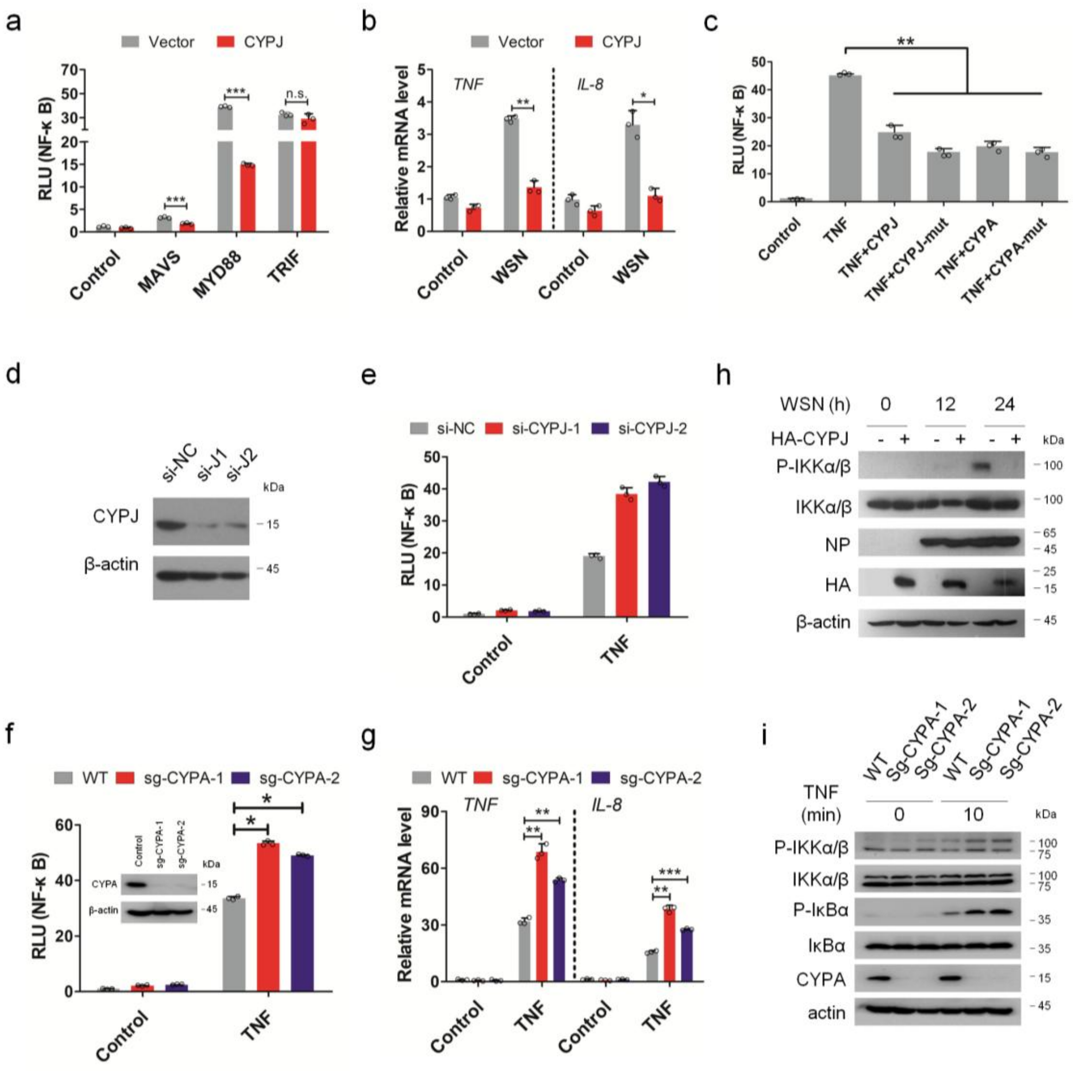

Supplementary Figure 1 | CYPJ negatively regulates NF-κB signal pathways. a, NF-κB reporter was cotransfected with plasmids encoding MAVS, MYD88 and TRIF with or without CYPJ in 293T cells, and the dual-luciferase activity was detected (N=3). **b,** CYPJ or control vector was transfected into 293T cells for 24 h, followed with WSN virus infection for another 24 h. The mRNA abundance of *TNF* and *IL-8* was evaluated by qPCR (N=3). **c,** NF-κB reporter

was cotransfected with plasmids encoding wildtype or catalytic death mutated (mut) CYPJ/CYPA in 293T cells, and the cells were treated with or without TNF (20 ng ml^{-1}, 5 h) followed with the detection of dual-luciferase activity. **d-e,** Short interference RNA (siRNA) mediated CYPJ knockdown (**d**) enhances TNF induced activation of NF-kB reporter in 293T cells as indicated (**e**; N=3). **f-g,** CRISPR-Cas9 mediated CYPA deficiency (**f**; inset) enhances TNF induced activation of NF-κB reporter in 293T cells (**f;** N=3). **g,** CYPA knockout enhances TNF induced transcription of TNF and IL-8 in 293T cells (**g**; N=3). **h,** 293T cells were transfected with control vector or HA-CYPJ for 24 h, followed with WSN infection for additional 12 h and 24 h. Whole cell lysates were prepared and analyzed by IB with indicated antibodies. **i,** Wildtype and CYPA-deficient 293T cells were treated with or without TNF for indicated time, and phosphorylation of indicated proteins were detected. Error bars indicate S.D.; n.s. no significance, *p<0.05, **p<0.01, ***p<0.001 (two-tailed Student's t-test).

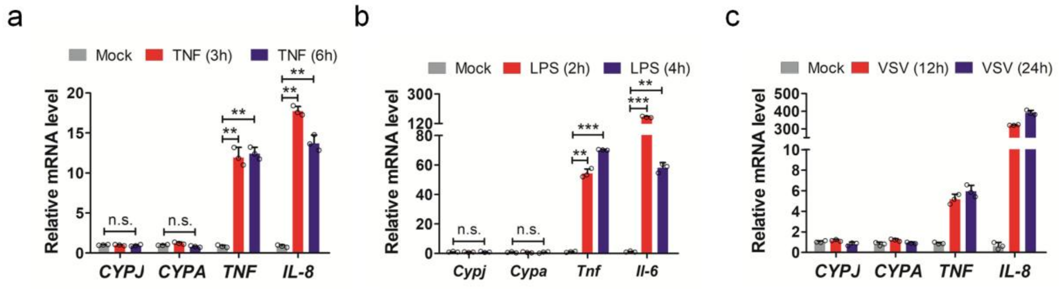

Supplementary Figure 2 | Transcription level of CYPJ and CYPA remain unchanged upon inflammatory stimulus. a-c, mRNA abundance of CYPJ/Cypj and CYPA/Cypa are elevated in TNF treated HeLa cells (**a**), LPS activated BMM cells (**b**) and VSV infected A549 cells (**c**) for indicated time. Error bars indicate S.D.; n.s. no significance, **p<0.01, ***p<0.001 (two-tailed Student's t-test).

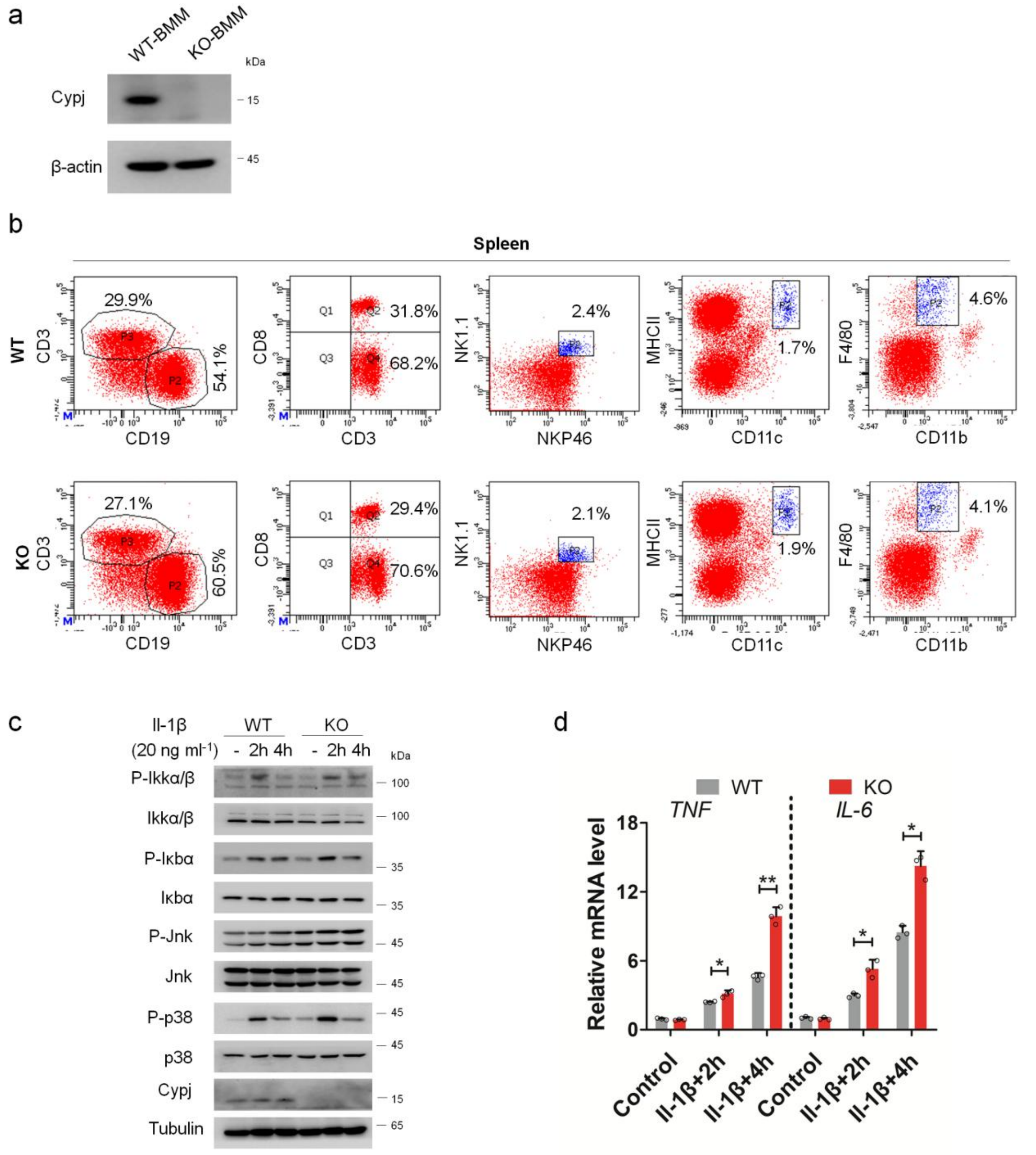

Supplementary Figure 3 | Cypj deficiency barely affects the development of immune cells. a, Protein level of endogenous Cypj in BMM cells from WT and KO mice were evaluated. **b,** Percentage of the indicated spleen immune cells was analyzed by detecting their specific lineage markers from WT and KO mice. One representative data from three independent experiments was

shown. **c-d,** Wildtype and Cypj-deficient primary mouse BMM cells were treated with or without Il-1β for different time, and phosphorylation of indicated proteins (**c**) as well as transcription of *Tnf* and *Il-6* (**d**; N=3) were detected. Error bars indicate S.D.; * p<0.05, ** p<0.01 (two-tailed Student's t-test).

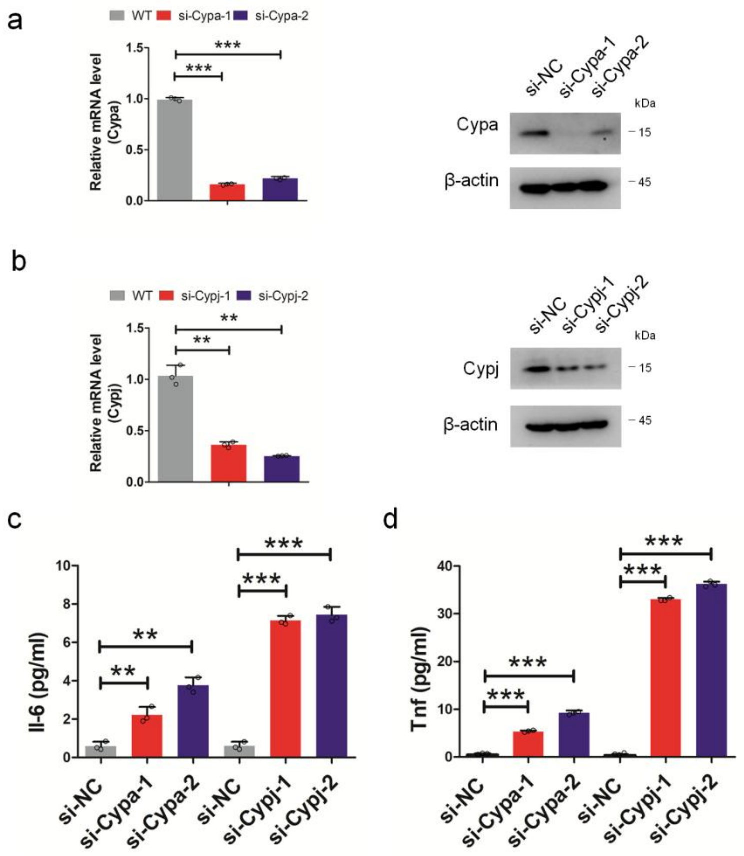

Supplementary Figure 4 | Silencing of Cypa and Cypj increase NF-κB activity. a, siRNA mediated Cypa knockdown efficiency in BMM cells were detected by qRT-PCR (**a**; left) and WB (**a**; right). **b,** siRNA mediated Cypj knockdown efficiency in BMM cells were detected by qRT-PCR (**b**; left) and WB (**b**; right). **c-d,** Secretion of Tnf (**c**) and Il-6 (**d**) in supernatant of BMM cells after siRNA transfection for 48 h were evaluated (N=3). Error bars indicate S.D.; ** $p<0.01$, *** $p<0.001$ (two-tailed Student's t-test).

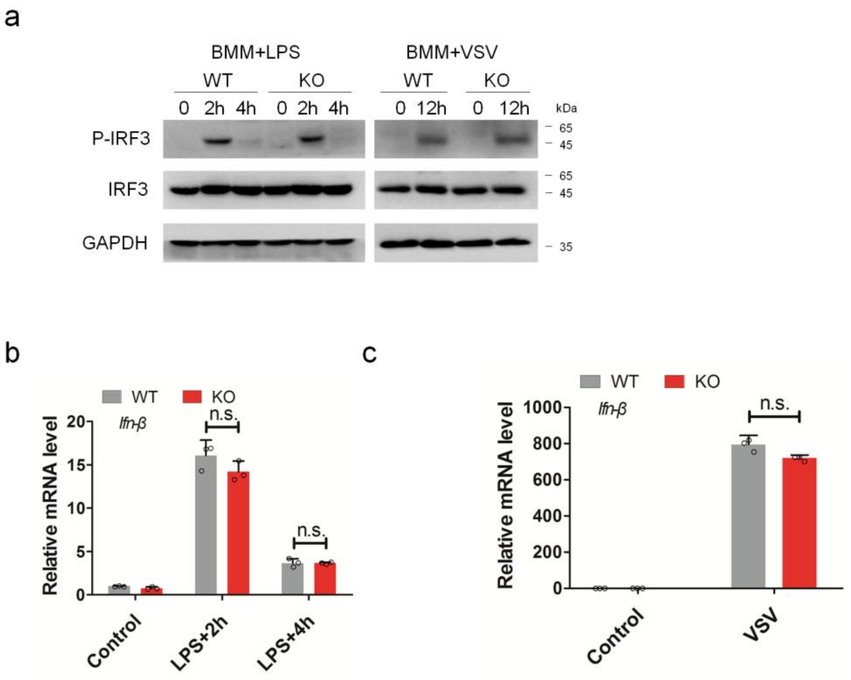

Supplementary Figure 5 | CYPJ does not affect the transcription of type I interferon. a, Wildtype and CYPJ deficient (KO) mBMM were treated with LPS (100 ng ml^{-1}) or infected with VSV (MOI=0.1) for indicated times, followed with the detection of IRF3 and P-IRF3 by WB. **b-c,** Wildtype and CYPJ deficient (KO) mBMM were treated with LPS (100 ng ml^{-1}) (**b**; N=3) or infected with VSV (MOI=0.1) (**c**; N=3) for indicated times, followed with the detection of *Ifn-β* transcription by qRT-PCR. Error bars indicate S.D.; n.s. indicates no significance (two-tailed Student's t-test).

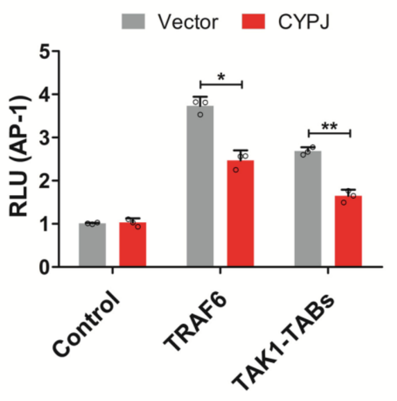

Supplementary Figure 6 | Effects of CYPJ on the activation of AP-1 reporter induced by TRAF6 and TAK1-TAB1/2. Error bars indicate S.D.; * p<0.05, ** p<0.01 (two-tailed Student's t-test).

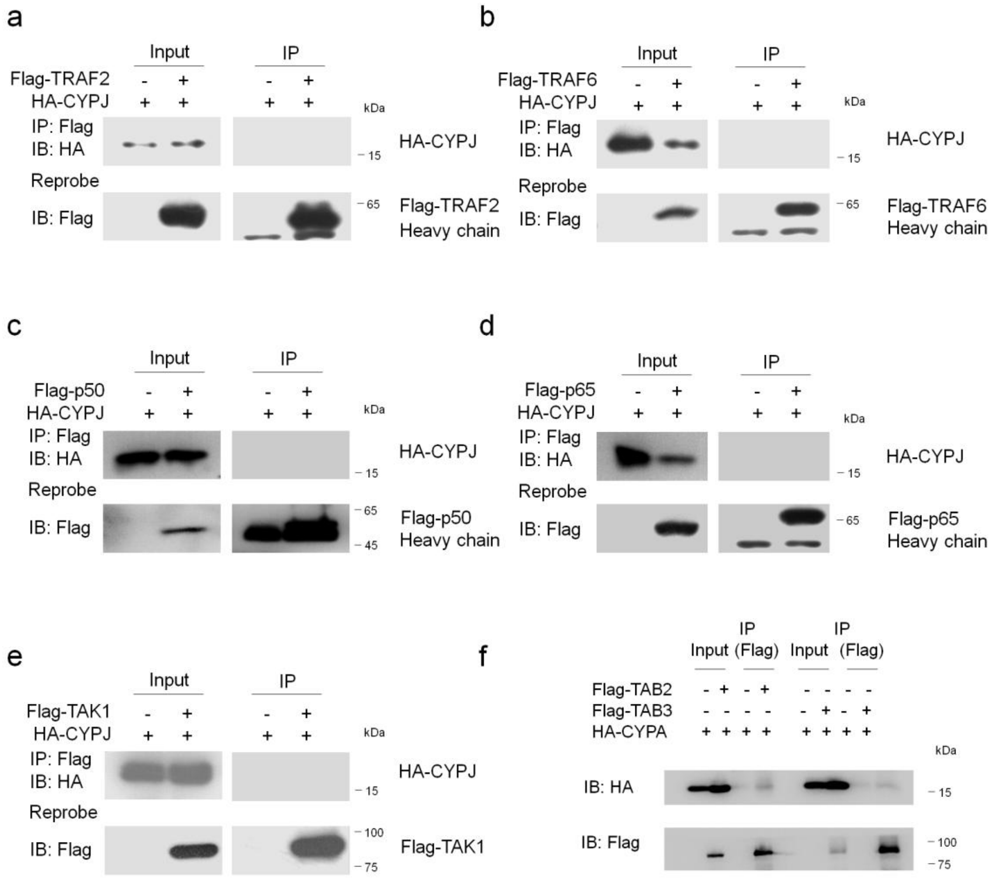

Supplementary Figure 7 | Screen the binding of CYPJ to several NF-κB components. a-e, Co-IP assays detecting the binding of HA-CYPJ to a series of Flag-tagged proteins of NF-κB signal pathway in 293T cells as indicated. CYPJ could not interact with TRAF2 (**a**), TRAF6 (**b**), p50 (**c**), p65 (**d**) or TAK1 (**e**). **f,** Co-IP assay showed that HA-CYPA could not interact with Flag-TAB2/3.

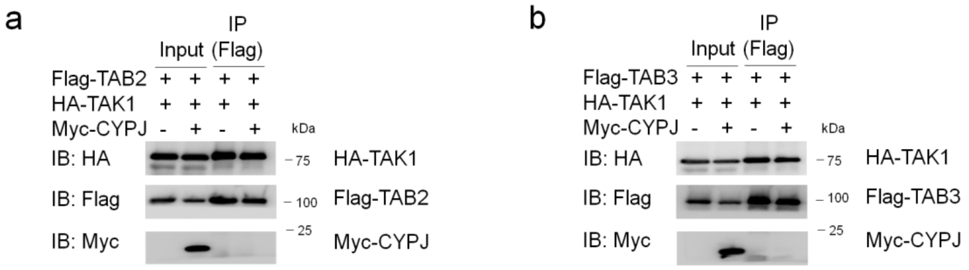

Supplementary Figure 8 | CYPJ does not affect the interaction between TAK1 and TAB2/3.

a-b, Co-IP assay detecting the binding between TAK1 and TAB2 (**a**) or TAB3 (**b**) with/without Myc-CYPJ co-transfection.

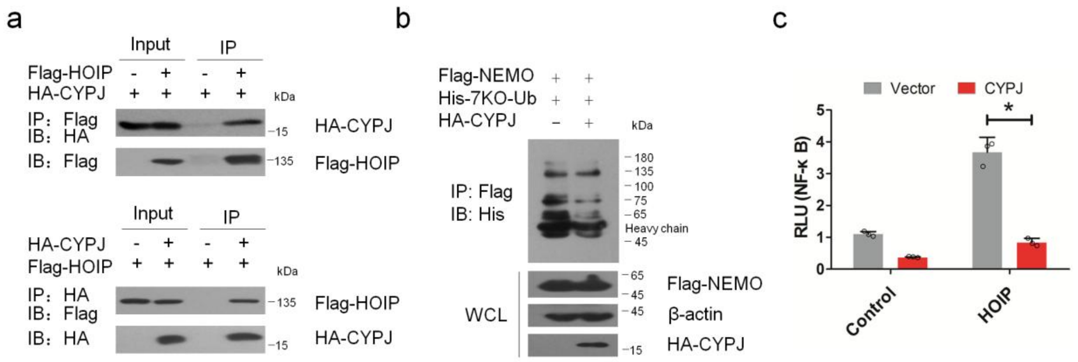

Supplementary Figure 9 | CYPJ interacts with HOIP and attenuates LUBAC activity. a, reciprocal Co-IP assay detecting the interaction between HA-CYPJ and Flag-HOIP. **b,** 293T cells were transfected with plasmids as indicated for 24 h. The enforced expressed NEMO was immunoprecipitated with anti-Flag antibody and its linear polyubiquitination was detected with anti-His antibody. **c,** NF-κB reporter was cotransfected with different combination of plasmids encoding HOIP and CYPJ in 293T cells, and the dual-luciferase activity was evaluated. * p<0.05 (two-tailed Student's t-test).

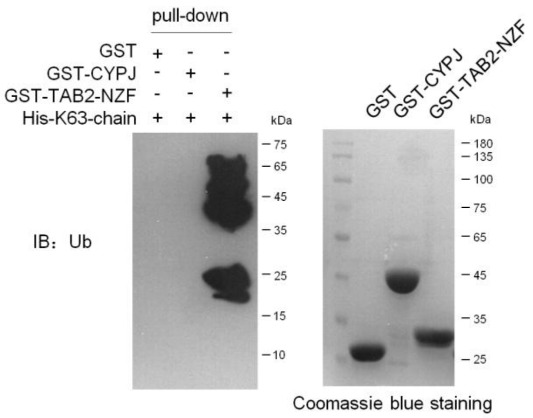

Supplementary Figure 10 | CYPJ itself could not bind ubiquitin chain. GST pull-down assay detecting the binding between GST, GST- CYPJ and GST-TAB2-NZF with His-K63-chain (2-7Ub). 1 μg of His-K63-chain (2-7Ub) is used in each reaction.

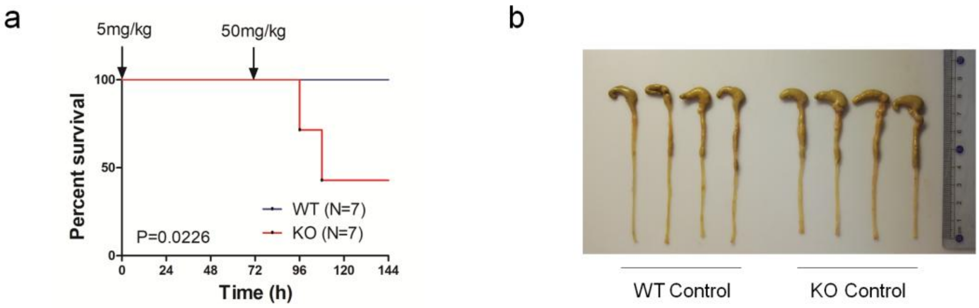

Supplementary Figure 11 | Cypj-deficient mice were sensitive to LPS treatment. a, Survival curve of wildtype and Cypj-deficient mice after intra-peritoneal injection of low dosage of LPS (5 mg per kg body weight) at day 0 following by high dosage of LPS (50 mg per kg body weight) at day 3 (N=7 mice per group; Log-rank test). **b,** Morphology of colon between wildtype and Cypj-deficient without 3% DSS treatment (N=4 mice per group).

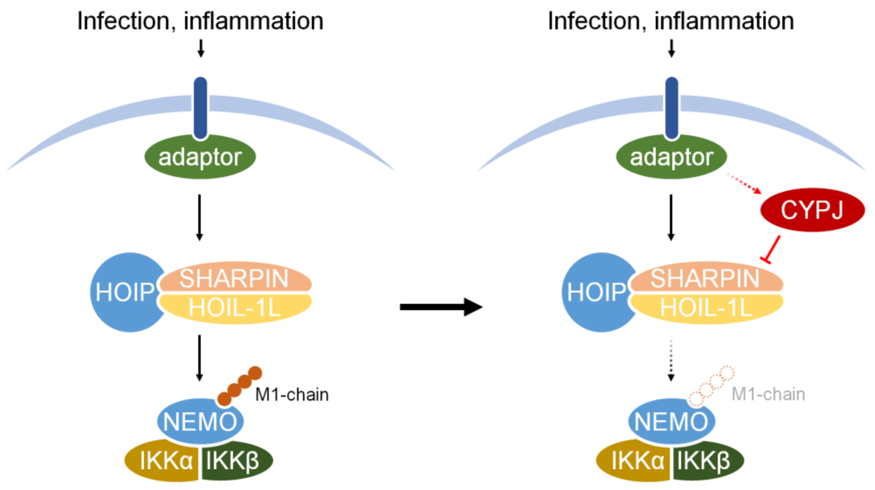

Supplementary Figure 12 | Proposed working model of CYPJ attenuates linear ubiquitin chain.

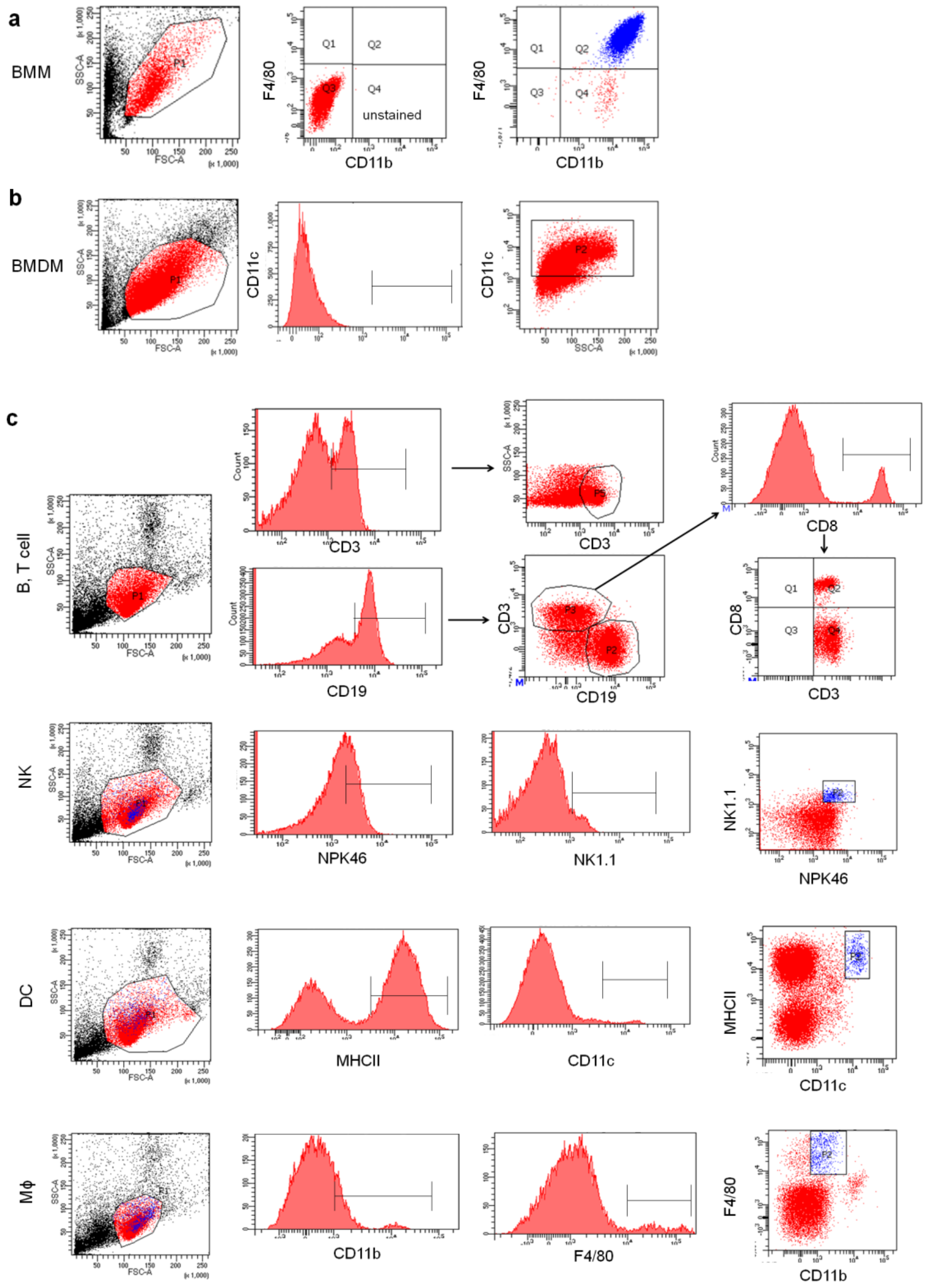

Supplementary Figure 13 | Gating Strategies used for immune cells analyses. a-b, Gating strategies to identify BMM (**a**) BMDM (**b**) used in Figures 2b/c. **c,** Gating strategies to indentify B, T, NK, DC and Mϕ lymphocytes in spleen used in Supplementary Figure 3b.

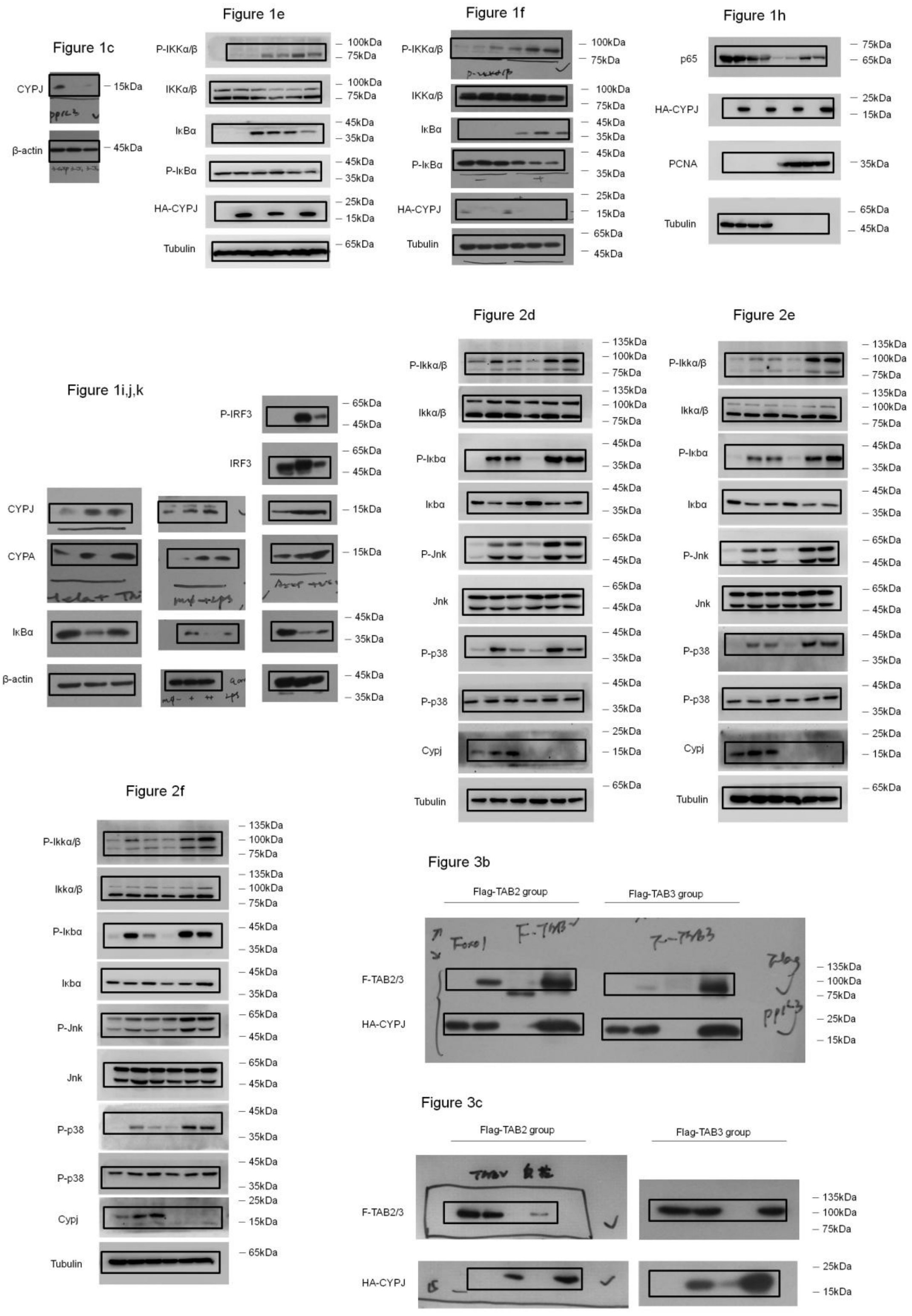

Supplementary Figure 14 | The uncropped scans of blots.

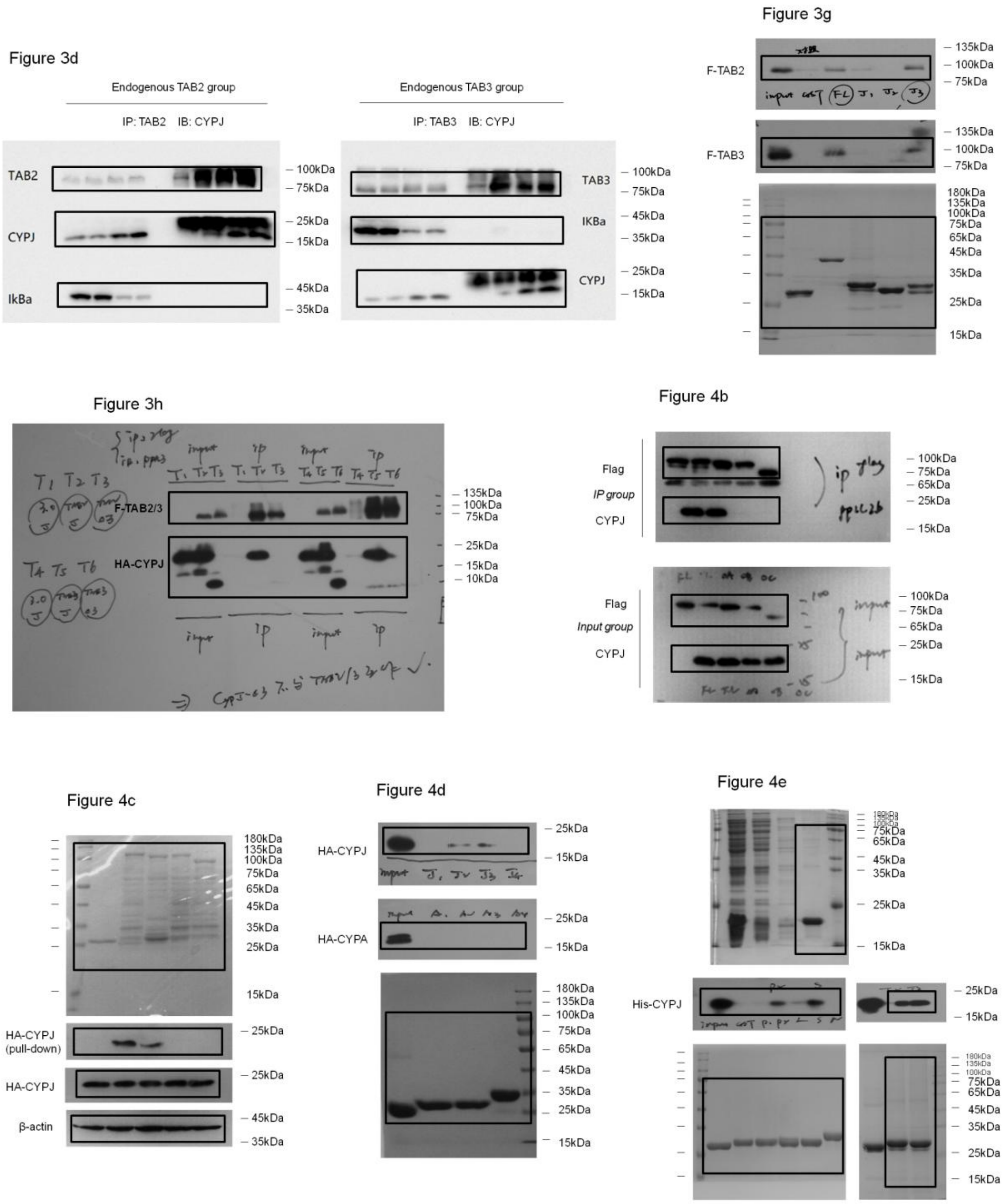

Supplementary Figure 14 | The uncropped scans of blots continued.

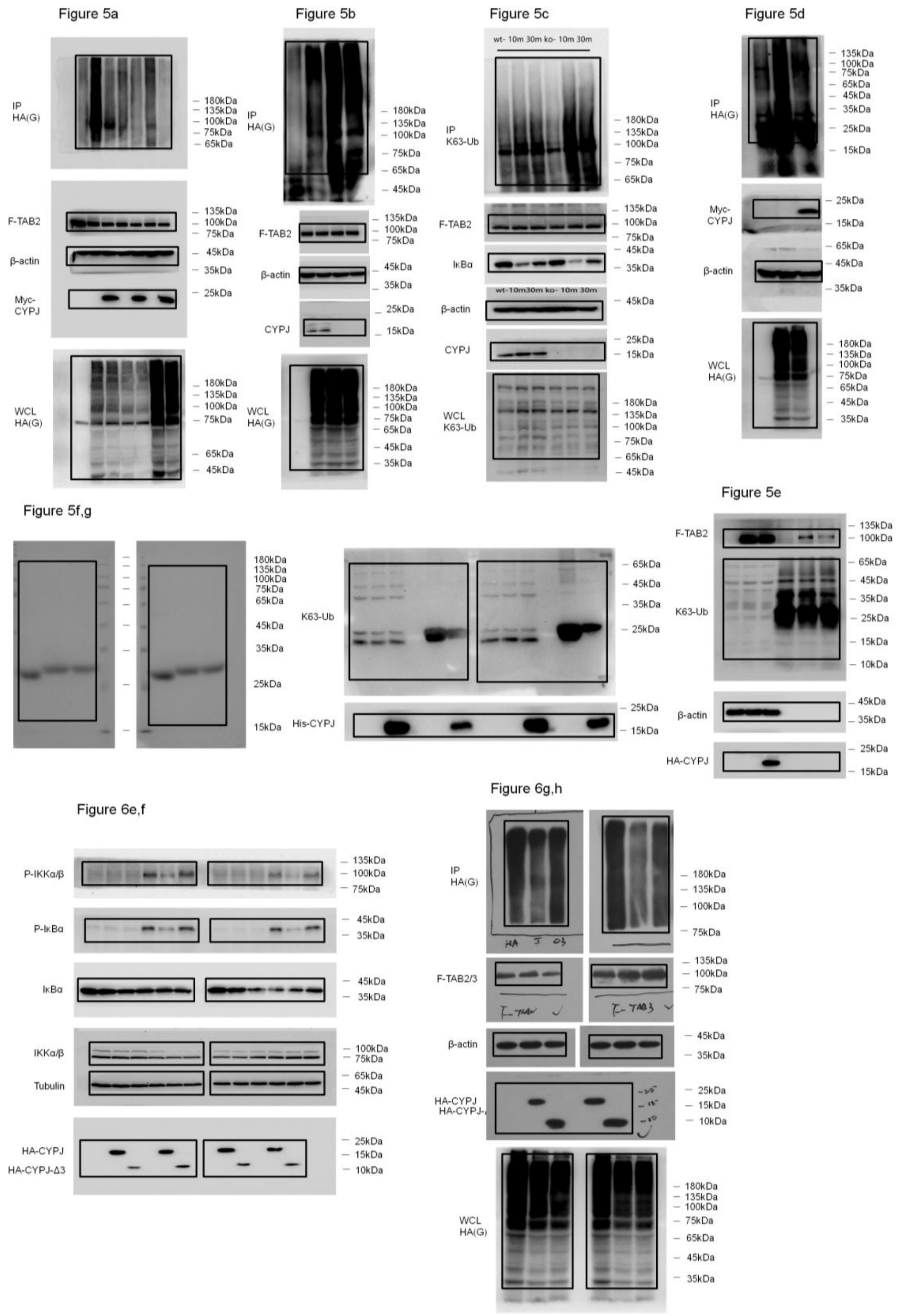

Supplementary Figure 14 | The uncropped scans of blots continued.

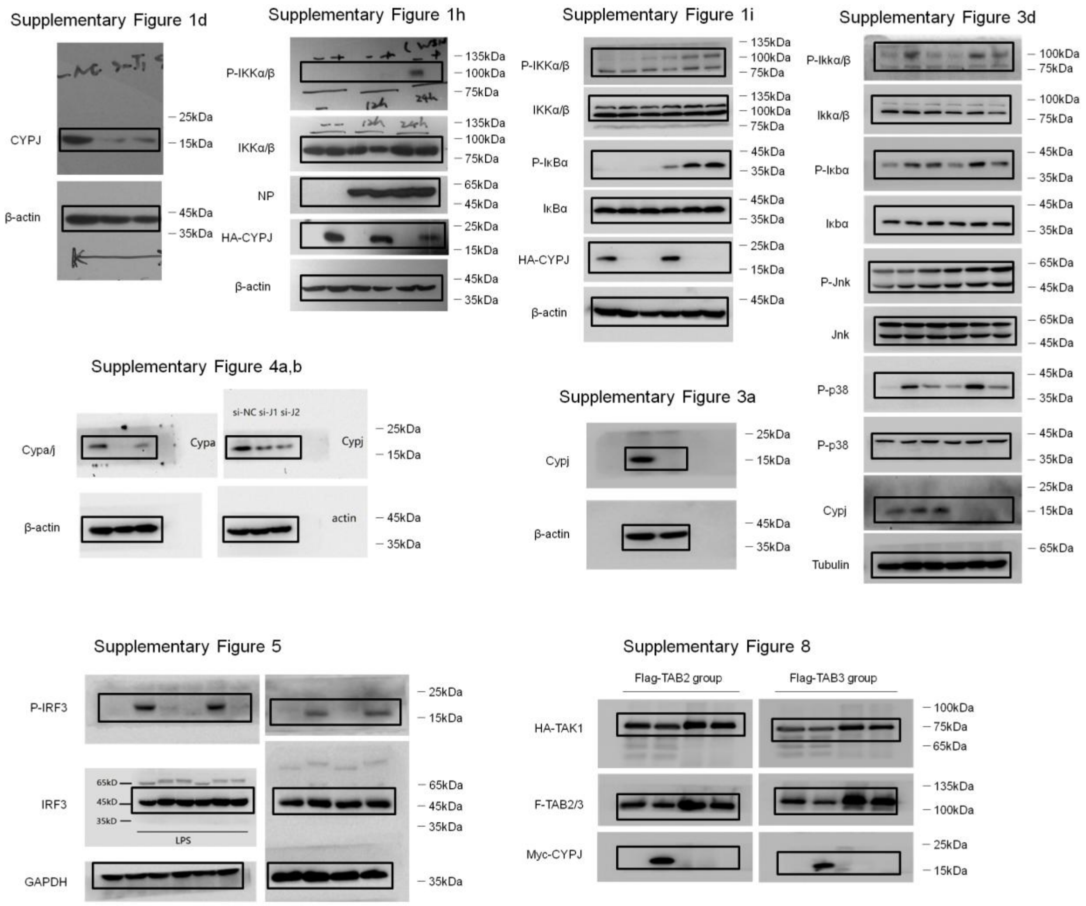

Supplementary Figure 14 | The uncropped scans of blots continued.

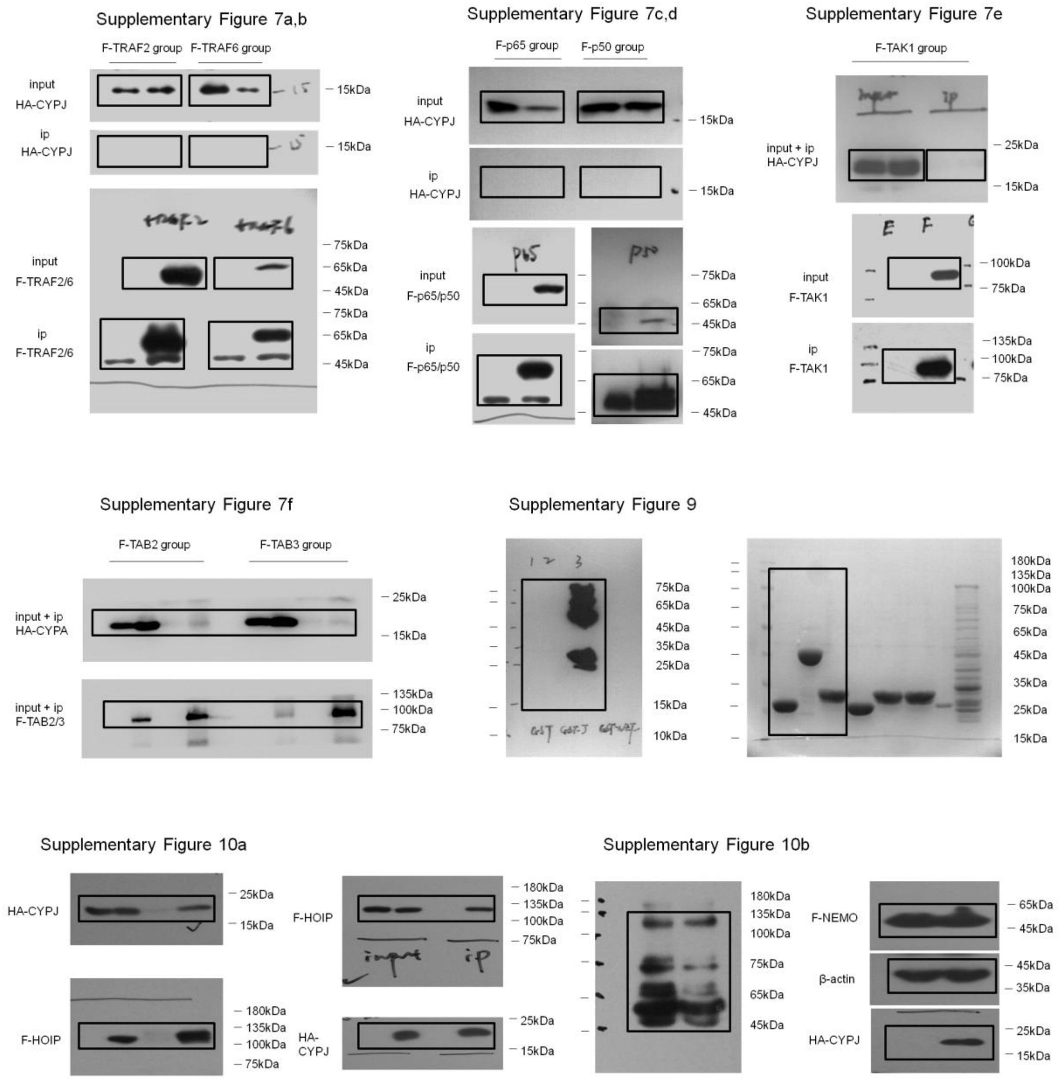

Supplementary Figure 14 | The uncropped scans of blots continued.

Supplementary Tables

Supplementary Table 1 | Primers for real-time qRT-PCR.

Gene name	Primer sequences (5'-3')
IFN-β	Forward: GTCAGAGTGGAAATCCTAAG<br>Reverse: ACAGCATCTGCTGGTTGAAG
TNF	Forward: AGTGAAGTGCTGGCAACCAC<br>Reverse: GAGGAAGGCCTAAGGTCCAC
IL-8	Forward: CTGCGCCAACACAGAAATTAT<br>Reverse: CATCTGGCAACCCTACAACAG
IL-6	Forward: GCCTTCTTGGGACTGATGCT<br>Reverse: CTGCAAGTGCATCATCGTTGT
CYPJ	Forward: CATCACCTATGGCAAACAGC<br>Reverse: TGGCAACTTCTCCAACTCATC
CYPA	Forward: AACTTCATCCTAAAGCATACGG<br>Reverse: TTGCCATCCAACCACTCAG
GAPDH	Forward: GGAGAAACCTGCCAAGTATG<br>Reverse: TTACTCCTTGGAGGCCATGTAG
Ifn-β	Forward: ATGAGTGGTGGTTGCAGGC<br>Reverse: TGACCTTTCAAATGCAGTAGATTCA
Tnf	Forward: AAGCCTGTAGCCCACGTCGT<br>Reverse: GGCACCACTAGTTGGTTGTCTT
Il-6	Forward: TAGTCCTTCCTACCCCAATTTCC<br>Reverse: TTGGTCCTTAGCCACTCCTTC
Cypj	Forward: CTGTGAGAGAACACCCAAAACA<br>Reverse: TTTTTGGCCCAGATGCTGCTA
Cypa	Forward: GAGCTGTTTGCAGACAAAGTTC<br>Reverse: CCCTGGCACATGAATCCTGG
Actin	Forward: AGTGTGACGTTGACATCCGT<br>Reverse: GCAGCTCAGTAACAGTCCGC
